# Urban settings do not ensure access to services: findings from the immunisation programme in Kampala Uganda

**DOI:** 10.1186/1472-6963-14-111

**Published:** 2014-03-06

**Authors:** Juliet N Babirye, Ingunn MS Engebretsen, Elizeus Rutebemberwa, Juliet Kiguli, Fred Nuwaha

**Affiliations:** 1 School of Public Health, Makerere University College of Health Sciences, P.O. Box 7072, Kampala, Uganda; 2 Centre for International Health, University of Bergen, Bergen, Norway

**Keywords:** Urban, Immunisation, Health system, Barriers, Resources, Service delivery, Public health, Mixed methods

## Abstract

**Background:**

Previous studies on vaccination coverage in developing countries focus on individual- and community-level barriers to routine vaccination mostly in rural settings. This paper examines health system barriers to childhood immunisation in urban Kampala Uganda.

**Methods:**

Mixed methods were employed with a survey among child caretakers, 9 focus group discussions (FGDs), and 9 key informant interviews (KIIs). Survey data underwent descriptive statistical analysis. Latent content analysis was used for qualitative data.

**Results:**

Of the 821 respondents in the survey, 96% (785/821) were mothers with a mean age of 26 years (95% CI 24–27). Poor geographical access to immunisation facilities was reported in this urban setting by FGDs, KIIs and survey respondents (24%, 95% CI 21–27). This coupled with reports of few health workers providing immunisation services led to long queues and long waiting times at facilities. Consumers reported waiting for 3–6 hours before receipt of services although this was more common at public facilities. Only 33% (95% CI 30–37) of survey respondents were willing to wait for three or more hours before receipt of services. Although private-for-profit facilities were engaged in immunisation service provision their participation was low as only 30% (95% CI 27–34) of the survey respondents utilised these facilities. The low participation could be due to lack of financial support for immunisation activities at these facilities. This in turn could explain the rampant informal charges for services in this setting. Charges ranged from US$ 0.2 to US$4 and these were more commonly reported at private (70%, 95% CI 65–76) than at public (58%, 95% CI 54–63) facilities. There were intermittent availability of vaccines and transport for immunisation services at both private and public facilities.

**Conclusions:**

Complex health system barriers to childhood immunisation still exist in this urban setting; emphasizing that even in urban areas with great physical access, there are hard to reach people. As the rate of urbanization increases especially in sub-Saharan Africa, governments should strengthen health systems to cater for increasing urban populations.

## Background

Three years into the decade of vaccines, 1.5 million child deaths occurred in one year due to vaccine preventable diseases [1] mainly in resource-limited settings. These accounted for 29% of all deaths among children aged 1–59 months [1] and occurred amidst unprecedented advances in vaccine technology and availability of new vaccines globally [2]. If rolled-out effectively, these advances and new vaccines could contribute significantly to accomplishing Millennium Development Goal (MDG) four: a two thirds reduction in childhood mortality. However, the lack of strong health systems necessary for their delivery might not allow the full impact of these interventions to be realised [3].

Uganda has recorded improved coverage for the third dose of the pentavalent vaccine comprising diphtheria, tetanus toxoid, pertussis, hepatitis B and *Haemophilus influenzae* (DPT3) from 46% in 2000 to 72% coverage in 2011 [4,5]. Coverage for DPT3 is considered a good indicator of health system performance. However, coverage estimates alone do not constitute a sufficient criterion for determining the achievement of certain performance levels by an immunisation programme [6]. Moreover, the coverage in 2011 fell short of GAVI targets of 80% DPT3 coverage in 80% of Ugandan districts. This continued failure to meet agreed targets suggests that specific challenges regarding immunisation programmes have not been fully identified, understood, or addressed [7,8]. Previous research on low vaccination coverage has focused on individual-level [8-14] and community-level factors [8,9,12,15]. A few studies, mostly in high vaccination settings, have examined immunisation services [13,16-20].

In the second goal of its 2011–2015 strategy [21], GAVI Alliance and other development partners are committed to making health systems in developing countries more effective in the delivery of vaccines. This cannot be achieved without understanding and addressing immunisation system barriers. This study assessed health system barriers to childhood immunisation services in Kampala the largest urban area in Uganda using consumer and provider perspectives. The WHO health system framework [22] was employed to analyse and present the findings using four of the building blocks: service delivery, human resource, finances and supplies, vaccines and technologies.

### The Ugandan immunisation system

The management of immunisation services in Uganda can be categorized into four subsystems, namely: immunisation management, vaccines management, health care service and community subsystems [15]. The immunisation management subsystem develops policy and standards in addition to management and monitoring of immunisation services at the national level. The Uganda National Expanded Programme on Immunisation (UNEPI) is charged with this responsibility [15,23]. The vaccines management subsystem delivers vaccines to the healthcare service subsystem at the district level. Previously, UNEPI was in charge of purchasing and delivering vaccines in Uganda. This role has been transferred during the past three years to the National Medical Stores, another semi-autonomous government-run organization. In the vaccines sub-system, there are sub-stores at the district and health sub-district levels before vaccines are delivered to peripheral health facilities [15,23]. Management in the districts disseminates UNEPI policy and standards, ensures maintenance of the cold chain, pays allowances to outreach personnel, conducts support supervision, receives and analyzes EPI data and gives feedback to UNEPI [23]. Management of the health facility delivers routine services to consumers at the health facility or during outreach activities; manages health workers, vaccines and equipment; provides health education; analyses data and submits monthly reports to the district [15,23]. The community subsystem represents the consumers of immunisation services [15]. This study examined health care service and community sub-systems.

## Methods

**Figure 1:**
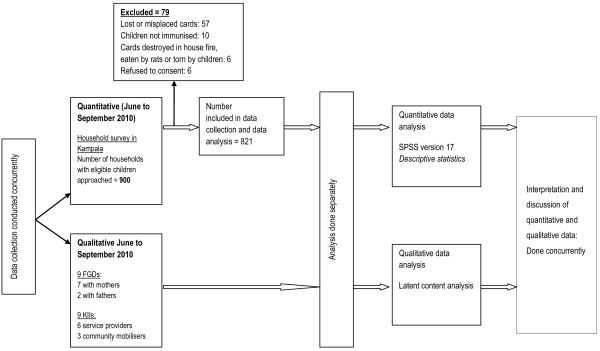
Study design.

A mixed methods approach was employed in which quantitative and qualitative data were collected using a concurrent triangulation design [24] as illustrated in Figure 1. By combining quantitative and qualitative data we sought convergence and corroboration among the different health system actors. The multiple perspectives provided an opportunity to develop a more complete understanding of the barriers to immunisation service delivery. The quantitative and qualitative data were analysed separately and integrated during interpretation of the results.

### Study setting

The study was conducted in Kampala from June to September 2010. The study setting was described in a previous publication [25] and only a brief description is provided here. Kampala is the capital city and the largest urban area in Uganda. It has a population of about 1.6 million. Children under 5 years constitute 20% of the total population. Kampala records the lowest childhood mortality rates in Uganda of 47 deaths per 1000 live births in 2011 [4].

Immunisation services in Kampala are provided by public, non-governmental organization (NGO) and privately owned health facilities. All public and NGO health facilities provide outreach services in addition to fixed/static routine immunisation services.

### Quantitative study

Quantitative data were collected in a cross-sectional design described elsewhere [25]. Briefly, we employed cluster sampling methods with a village as a cluster. The required sample size was 812 households using the formula by Bennnet et al. [26] for cluster surveys with the following assumptions; a two-sided test with a precision of 0.03, 80% power, 7 households per cluster, intraclass correlation of 0.1, design effect of 1.6, proportion of those with complete vaccinations 47%, and a non-response rate of 37% (estimated among children aged 12–23 months with missing child health cards) [27].

#### ***Sampling***

The sampling technique employed in this study comprised two stages. In the first stage, parishes were randomly selected using computer-generated numbers, resulting in 10 selected parishes out of 44 in the Nakawa and Makindye divisions. The number of respondents in each parish was determined using sampling proportionate to the number of infants in that parish. All villages in the parish were included in the study. In the second stage, households were selected consecutively starting from the house on the eastern side of the main junction in the village and moving in clock-wise concentric circles around the starting point until the sample for the village was obtained. One child caretaker per household was selected. Child caretakers were eligible for study inclusion if they were from households with a child aged 10 to 23 months and if they had a child health card. This last criterion was chosen to reduce recall bias of the primary outcome of the study. If a respondent in a selected household had no eligible child, declined to participate, was less than 18 years of age or was not at home when the house was approached for study inclusion, the next household was considered for study inclusion.

#### ***Quantitative data collection and analysis***

An interviewer-administered questionnaire was employed to collect data on the socio-demographic and economic characteristics and on questions derived from the WHO health systems framework [22] specifically from the following building blocks: service delivery, human resource, finances and supplies, vaccines and technologies. The questions included self-reported distance to the immunisation facility, choice of facility for immunisation services, waiting time before receipt of immunisation services, reception by service providers, cost of immunisation services, complications experienced after vaccination e.g. fever or injection abscess, missing of immunisation appointments, and whether any of these issues would prevent study participants from seeking immunisation services.

The household wealth index was developed using principal components analysis [28] with variables on asset ownership (radio, telephone, television, refrigerator, cupboard, bicycle, motorcycle, car/truck); structural materials of the dwelling (floor, wall, roof); availability of electricity, water and sanitation services; number of rooms in the house; and house ownership. The first component explained 30.9% of the variance. Regression factor scores generated from the first principal component were ranked in ascending order and then categorised into quintiles (1) poorest, to (5) least poor.

Mobile phones were used to collect the data. The questionnaire was designed and managed using OpenXdata version 1.3.4 (http://www.openxdata.org). The questionnaire was uploaded to mobile phones and the collected data were synchronized to a database on a daily basis via the internet. The data saved on the server were exported to Excel and SPSS, v. 17 (SPSS Inc. Chicago, Illinois) for analysis. Statistical data analysis employed descriptive statistics using proportions with their 95% confidence intervals. Use of immunisation services from public or private facilities were the major dependent variable, since it was found that private facilities were engaged in immunisation service provision two years prior to the study. Therefore, univariable and multivariable logistic regression analysis was conducted for this dependent variable in relation to the relevant variables from the WHO building blocks. Cluster sampling was adjusted for in all analyses using complex samples analysis employing the probability proportional to size sampling method. All variables with a p value ≤ 0.1 at univariable analysis were entered into a multivariable model and model robustness was checked by Wald chi square.

### Qualitative study

Qualitative data were collected using focus group discussions (FGDs) and key informant interviews (KIIs). The methods used for the FGDs are presented elsewhere [9]. Overall, 9 FGDs were held among 58 women and 15 men: three were with mothers aged 18–25 years, four with mothers older than 25 years and two with fathers.

Nine KIIs were held with six health providers and with three of those in charge of community mobilization for immunisation. The health providers included two focal persons for immunisation management at the district level, three mid-wives in charge of immunisation at three health units and one nurse in the district vaccine store. Two of the KIIs with those- in- charge of community mobilization were conducted in Luganda (local language); the rest were conducted in English. The KIIs with health providers were conducted by JNB and the KIIs with those in charge of community mobilisation were conducted by a research assistant.

The KII and FGD guides focused on perceptions and experiences with barriers to childhood immunisation services, their causes, and local solutions to these problems. The number of FGDs/KIIs was deemed sufficient when additional interviews yielded little new information on the core study objectives.

#### ***Qualitative data management and analysis***

All the data were tape-recorded after obtaining the participants’ consent. The audio data were transcribed verbatim and those in the local language were translated into English after transcription by the moderator. JNB listened to the audio recordings to confirm the information on the transcripts. The unit of analysis was the transcripts from FGDs and KIIs according to Granheim and Lundman [29]. Data were analysed by latent content analysis [29]. This process entailed the authors reading through the transcripts and discussing the content. Meaning units were generated from the text and condensed into codes. The authors sometimes identified different issues, and during the debate that ensued we eventually proposed codes that were discussed and agreed upon. The authors went back to code again using the agreed codes and these were merged into categories and then into themes. The themes were grouped and presented according to the WHO health system framework [22]. The different data sources informed each other during design, implementation and qualitative data analysis [30].

### Ethical approval

Ethics approval was obtained from Makerere University School of Public Health Higher Degrees Research and Ethics Committee (IRB00005876FWA/Protocol 085) and from the Uganda National Council for Science and Technology (HS 786). This study complied with ethical guidelines for research using human subjects and the interviews or discussions were conducted only when informed and written consent had been obtained from the study participants.

## Results

This section presents integrated quantitative and qualitative findings under four WHO health system building blocks: service delivery barriers (including geographical access and quality of immunisation services); human resource barriers; lack of supplies and transport; and financial barriers to service usage. The theme on quality of services is further divided into two sub-themes: safety during immunisation services and waiting time before receipt of services. Finally, the theme on human resource barriers presents findings on the number and attitudes of service providers. In the survey there were 122 clusters with a total of 821 respondents, 96% (785/821) of whom were mothers with a mean age of 26 years (95% CI 24–27).

### Service delivery

In this sub-section, we present findings on the service delivery building block under two sub-themes: geographical access to immunisation services and quality of immunisation services.

#### ***Geographical access to services***

All data sources reported that there was poor distribution of facilities that provide routine childhood immunisation services, that is, almost all FGDs and all key informants. This finding was corroborated by the survey in which a quarter (24% 95% CI 21–27) of the respondents reported living more than 2 km from an immunisation facility. Of these, 74% (95% CI 67–80) received services from public facilities and 26% (95% CI 20–33) from private facilities. There was no statistical association between distance to the immunisation facility and whether immunisation services were received from public or private facilities (OR 0.73, 95% CI 0.39-1.34; Table 1).

**Table 1:** Barriers to immunisation services by public and private immunisation facilities (Cluster adjusted)

**Varriable**	**Proportion of total**	**Immunisation facility**		**Unadjusted**	**Adjusted**
	**% (95% CI)**	**Public n (%)**	**Private n (%)**	**OR (95% CI)**	**OR (95% CI)**
**Distance to the immunisation facility**					
<2 km	76 (73–79)	399 (73)	197 (79)	0.73 (0.39-1.34)	
>2 km	24 (21–27)	145 (27)	52 (21)	1	
**Household wealth index**					
Top quintile, Least poor	20 (17–23)	93 (17)	64 (27)	1	1
4th quintile	20 (17–23)	122 (22)	38 (19)	2.21 (1.51-3.22)	1.49 (0.77-2.81)
3rd quintile	19 (16–22)	102 (19)	47 (20)	1.49 (0.57-3.88)	2.11 (0.96-4.65)
2nd quintile	21 (18–24)	112 (21)	50 (21)	1.56 (0.74-3.29)	1.35 (0.59-3.11)
Bottom quintile, poorest	20 (17–23)	116 (21)	42 (17)	1.89 (0.93-3.86)	2.81 (1.58-5.00)
**Place of delivery**					
Hospital	63 (60–67)	376 (68)	134 (54)	1	1
Health centre	30 (26–33)	138 (25)	100 (40)	0.49 (0.35-0.68)	0.64 (0.27-1.49)
Home	7 (4–9)	40 (7)	16 (6)	0.88 (0.45-1.72)	0.74 (0.19-2.89)
**Duration participants are willing to wait for services**					
<1 hour	43 (39–46)	236 (43)	101 (40)	1	
1-3 hours	24 (21–27)	130 (23)	65 (26)	0.86 (0.48-1.54)	
>3 hours	33 (30–37)	188 (34)	84 (34)	0.97 (0.50-1.85)	
**Has ever missed an immunisation appointment**					
Yes	44 (41–47)	234 (42)	119 (48)	1	
No	56 (53–59)	321 (58)	131 (52)	1.38 (0.69-2.75)	
**Reason for missing appointment**					
Caretaker related	73 (68–78)	164 (70)	90 (77)	1	
Child related	27 (22–32)	69 (30)	27 (23)	1.42 (0.81-2.47)	
**Developed abscess after immunisation**					
Yes	6 (4–8)	36 (7)	17 (7)	1	
No	94 (92–96)	519 (93)	233 (93)	1.06 (0.52-2.21)	
**Developed fever after immunisation**					
Yes	43 (40–47)	214 (39)	138 (55)	1	1
No	57 (53–60)	341 (61)	112 (45)	1.96 (1.37-2.79)	1.08 (0.49-2.38)
**Sought care after child developed fever**					
Yes	89 (86–92)	189 (88)	125 (91)	1	1
No	11 (8–14)	25 (12)	13 (9)	1.25 (0.64-2.43)	1.31 (0.75-2.29)
**Would be hindered if health worker were rude**					
Yes	25 (22–28)	138 (25)	63 (25)	0.97 (0.62-1.53)	
No	75 (72–78)	416 (75)	185 (75)	1	
**Do you incur costs for immunisation**					
Yes	62 (59–65)	324 (58)	176 (70)	1	1
No	38 (35–41)	231 (42)	74 (30)	1.69 (1.02-2.82)	0.60 (0.37-1.00)
**Do you incur transport costs while seeking immunisation services**					
Yes	45 (41–48)	262 (47)	101 (40)	1.32 (0.83-2.08)	
No	55 (52–59)	293 (53)	149 (60)	1	
**Transport costs would hinder seeking immunisation services**					
Yes	30 (26–33)	164 (30)	74 (30)	1.01 (0.63-1.60)	
No	70 (67–74)	391 (70)	176 (70)	1	

As reported by participants during all FGDs, in some places there was only one facility to serve many villages (administrative units) which were densely populated. This poor distribution of services sometimes deterred parents from taking their children for immunisations. A 40 year old father of five from a densely-populated neighbourhood emphasized:

*“The major problem we experience is that the (immunisation) services are very far. And so some people go (for immunisation) and others don’t because the distance is long and that makes our children get problems.”* Male FGD

However, 74% (95% CI 71–77) of the respondents in the survey reported that the long distance to the immunisation facility would not deter them from using immunisation services.

The poor distribution also resulted in long queues at the facility despite daily provision of immunisation services. The immunisation providers and managers reiterated that distribution of immunisation services was poor. They argued that consumers did not want to travel for more than 1 km to seek services but preferred services closer to their residences. This preference was attributed to several contextual and environmental issues such as poor road networks and limited access to public transport.

The managers added that in the two years prior to this study they had addressed the issue of geographical access using a three pronged approach: first, they remapped all outreach immunisation services by increasing the number of outreach posts; secondly, they increased the distance between some immunisation facilities that had previously been only half a kilometre apart; thirdly, they involved private-for-profit facilities in service provision. Engagement of the private sector in service provision was prompted by the perception that much of the population of Kampala sought health services from private facilities. This finding was corroborated by survey data revealing that 30% (95% CI 27–34) of the respondents reported receiving immunisation services from private facilities and 68% (95% CI 64–71) from public facilities. Only 2% of the respondents received immunisation from outreach immunisation sites. Overall, the managers’ perception was that the private sector still had high potential for service provision although its engagement remained low. However, the lack of financial support to private facilities could have hampered their full engagement in service provision. A manager of immunisation in Kampala emphasized that:

*“There are a lot of complaints by immunisation providers especially those in private facilities. They ask, ‘aren’t we supposed to be paid for this service?’ There should be some financial incentive because they look at immunisation like a special service.”* KII

#### ***Quality of services***

The other aspect of the service delivery building block considered was quality of immunisation services. We limit our examination to two sub-themes: safety during immunisation services and waiting time before receipt of services. When service providers and managers were asked about the quality of immunisation services most appeared surprised by the question. Some responded *‘the quality of service is good’* without qualifying this assertion. A few noted that the important issues in immunisation service provision were maintenance of vaccine viability and availability of vaccines.

Most key informants added that mostly *‘wealthier’* child caretakers sought services from private facilities. This finding was partly evident from the survey data although there was no linear relationship between wealth and choice of service providers, Table 1. The odds of using private facilities for immunisation services among respondents whose households were in the top quintile (least poor) were three times higher than those whose households were in the bottom quintile (poorest; OR 2.81, 95% CI 1.58-5.00). However, the findings from key informants and survey data on the choice of service provider were contradicted by participants from most female FGDs, who reported a preference for public facilities for immunisation. These facilities were perceived to be of better quality because they were believed to have ‘experts’ or ‘specialists’ in reference to the qualifications of the service providers.

##### 

**Waiting time** Participants from all FGDs and those in charge of community mobilisation reported long waiting times before receipt of immunisation services. In a FGD, a 50 year old father of six emphasized, ‘*instead of you taking 30 minutes you take about 6 hours before you get the services.’* Most private facilities provided services within 30 minutes of arrival at the facility. The respondents reported that long waiting times occurred mostly at public facilities and a few private facilities. A 52 year old female reported:

*“At (the public facility) you can even come back without receiving immunisation. You have to wait a long time, the children will cry (and yet) you have another child at the pre-school and it is coming to noon, and have to come back to pick her from the pre-school. You can come back without immunising the other one.”* FGD women

The providers and managers reiterated the reports of long waiting times and said this happened at public but not private facilities. They also said that delays were primarily due to health system delays e.g. delays in vaccine supply or travel time by providers, especially those conducting outreach immunisation services. The consumers reported similar reasons for the delays, as exemplified by the following quotation from one of those in charge of community mobilisation:

*“This delay occurs because some health facilities have no refrigerators to store the vaccines and have to send one of the health workers to the headquarters for vaccines. They delay to return and when they eventually arrive, it is quite late in the morning (past mid-morning).”* KII

For working mothers, the delays acted as barriers to service utilization since they could not access services in the time available before they reported to work. Some therefore resorted to paying up to 10,000 Uganda shillings (equivalent US$ 4) so they could receive the service from private facilities over the weekend. In contrast, a 24 year old mother disagreed with those whom she perceived as uncommitted to immunisation. She emphasized that the level of commitment required for seeking immunisation services was comparable to visiting a good hair dresser, which is a protracted event for an urban Ugandan woman.

“If you know that a good hair dresser is three kilometres away, even if you found very many people in the queue waiting to be served, you would wait for your turn without any complaint.”

Survey data complemented the qualitative findings; 43% (95% CI 39–46) of survey respondents said they were only willing to wait for up to an hour before receipt of services; 24% (95% CI 21–27) were willing to wait for 1–3 hours and 33% (95% CI 30–37) for more than 3 hours. More of those who had attained secondary education than those who had not completed primary education preferred receiving services within an hour of arrival at the immunisation facility (OR 2.09, 95% CI 1.2-3.62). The duration individuals were willing to wait for services was not associated with the respondent’s marital status, occupation or whether services were received from public or private facilities, Table 1.

##### 

**Safety** The other aspect of quality of services examined was safety of immunisation services. Safety of the children during immunisation was a major concern for most respondents in FGDs. They reported that some children developed injection abscesses following vaccination and that other providers caused injuries to the babies during the vaccination process. A 23 year old mother of two reported her observation:

*“One time I took my child for immunisation, a nurse injected another child on the thigh, the needle curved and a lot of blood came out. The mother (of the child) quarrelled with the nurse and the other mothers (present) joined her. They all protested and went out (without receiving immunisation services).”* Female FGD

The injection abscesses described by child caretakers were distinct from the abscesses that developed after a BCG injection on the upper arm of the child. About 6% (95% CI 4–8) of survey respondents reported abscess formation after vaccination. Nearly all (94%, 95% CI 90–98) suffered these abscesses for less than a week with a mean duration of 4.3 days (SD =4.0) and a median of three days. There was no statistical association between reporting abscesses after vaccination and receiving services from private or public facilities, Table 1 (OR 1.06, 95% CI 0.52-2.21). Of those that developed abscesses, 15% (95% CI 5–25) said they would not take their children for further immunisations and they would dissuade others from doing so.

The occurrence of fever after immunisation was reported among 43% (95% CI 40-47%) of survey respondents. Most (80%, 95% CI 70-90%) of these reported that the fever lasted for less than two days and only 4 children were admitted as a result. Respondents who received immunisation services from private facilities reported fever twice as commonly (OR 1.96, 95% CI 1.37-2.79) as those who received them from public facilities. This however did not remain significant at multivariable analysis. About 11% (95% CI 8-14%) of those whose children developed fever after immunisation did not seek care for the fever. Among those who did seek care, only 39% (95% CI 33-44%) sought it from health care workers. The rest used home remedies to treat the fever.

From the qualitative data, concerns for child safety during immunisation were reportedly linked to doubts among consumers about the qualifications of some of the service providers who conducted the immunisation services. The immunisation managers agreed there were unqualified people participating in the immunisation programme, but they were mainly in private facilities. They proposed that private facilities inevitably prioritized the most profitable services at the expense of the important but unprofitable immunisation activities, as demonstrated by their deployment of unqualified staff to run the latter. One of the managers of immunisation reported that:

*“These days you know we have a lot of nursing assistants in private health units. They are unqualified to provide immunisation. It’s a disaster!”* KII

### Availability of supplies and transport

Most consumers in almost all FGDs and those in charge of community mobilisation reported intermittent availability of vaccines. This poor supply of vaccines led to incomplete or untimely immunisations, loss of time and financial resources for the customers, and loss of faith in the immunisation service. A 26 year old mother of one expressed her disappointment in the following quotation:

*“You can even walk to and fro for two months to public hospitals and they tell you they don’t have vaccines. My child missed many times until I got fed up.”* Female FGD

Most providers and managers corroborated instances of vaccine shortages, especially polio vaccine, which was unavailable for up to one month in some facilities. However, they blamed ‘management at the head office’ for this problem.

Besides vaccine shortages, poor availability of transport was reported. Specifically, most health units visited had no vehicle designated for immunisation activities. Some, mostly providers at private facilities, used the ambulance for immunisation activities. Poor availability of transport was common at public and private health facilities and was cited by providers as a major hindrance to service provision. In addition, other private facilities had to find ways of collecting vaccines from the vaccine store since they did not obtain transport from the immunisation programme. A manager of immunisation reported:

*“The immunisation providers from private facilities have to use their own transport as they come to pick vaccines weekly. That money that they spend weekly to come for vaccines, they feel that it should be refunded.”* KII

Key informants said that it was difficult for providers who participated in outreach services to report to these facilities without a vehicle because they had to carry bulky immunisation boxes containing vaccines and the facilities were far from their usual work place. As an alternative to inadequate transport for all immunisation activities, the providers reported that the immunisation programme provided them with cash allowances so they could use public transport to report to the outreach facilities. However, these allowances were frequently delayed for up to three to six months.

### Human resource barriers

Under the human resources block we examined the number and attitudes of service providers.

#### ***Number of providers***

Dissatisfaction with the number of providers engaged in immunisation service provision was expressed by those in charge of community mobilisation and among most participants in a few female FGDs. A 27 year old mother of three reported that they usually found one or two providers in charge of immunisation. This report was corroborated by service managers who said that having at least one individual in service provision was acceptable for immunisation activities. However, they explained that only in private facilities were the numbers of personnel for immunisation inadequate. Users of immunisation services contradicted this, arguing that having one or two service providers for the many women who turned up for services was inadequate. This finding was not investigated in the survey.

High attrition of service providers due to internal migration to better-paying health facilities was reported to occur commonly among private facilities and some public facilities. This high attrition resulted in discontinuation or interruption of immunisation services.

#### ***Attitudes of providers***

In addition to reporting that there were few service providers, consumers reported poor attitudes among some of them. This was expressed by most mothers in all female FGDs. The participants in the male FGDs did not report it and most providers denied it. The poor attitude was reportedly manifested as *‘verbal abuse’,* ‘*poor or lack of communication with the consumers’.* Mothers reported that although they went to immunisation facilities early as instructed by providers, they often waited for services with no explanation for the delayed services. They also reported experiencing or observing ‘verbal abuse’ from service providers. The common reasons for this treatment ranged from *‘delaying to undress the child’* for vaccination to *‘missing previous vaccination appointments’*. Overall, 44% (95% CI 41–47) of survey respondents reported missing at least one appointment for immunisation. There was no statistical association between those who had ever missed appointments for immunisation and whether they received services from public or private facilities (OR 1.38, 95% CI 0.69-2.75), Table 1.

A few of the mothers in the FGDs felt they were in the wrong and therefore deserved to be admonished by the immunisation providers; others were puzzled by this behaviour. A 43 year old mother of five said, “*You will just wonder why she treated you like that because she does not know you. Even those around you will wonder why she is (verbally) abusing you.”* Some service users expressed fear of seeking immunisation services after being disrespected by the providers. This was also revealed by in the survey data. One quarter (25%, 95% CI 22-28%) of the survey respondents reported that they would be deterred from seeking immunisation services if health workers were rude. There was no statistical difference in the proportions that would be deterred by health worker behaviour among those who received services from public or private facilities (OR 0.97, 95% CI 0.62-1.53), Table 1. FGD participants reported that poor attitudes and verbal abuse from service providers were observed almost exclusively in public facilities and they branded this as health worker *‘culture’* since they behaved similarly in other departments of the health facility.

### Cost of immunisation services

Under the system building block of financing we assessed the cost of immunisation services to the consumers. All providers emphasized that immunisation services were free in both public and private facilities. However, all interviews or discussions with consumers revealed that people paid 500 to 10,000 Uganda shillings (equivalent of US$ 0.2 to US$ 4) for services. More than half (62%, 95% CI 59–65) of the survey participants reported incurring costs for vaccination. Costs were more commonly incurred at private facilities (70%, 95% CI 65–76) than at public facilities (58%, 95% CI 54–63; OR 1.69, 95%CI 1.02-2.82), Table 1.

The costs for immunisation were said to impact negatively on usage of immunisation services. A 20 year old mother of one reported, “*I had money only the first time and the child was immunised (that time). (But) the child has never been immunised since I failed to raise the money for subsequent doses”.* A minority of consumers were willing to pay for the services since they reasoned that the benefits of immunisation outweighed the cost. In contrast, most respondents from more than half the female FGDs were bitter about being made to pay for services that were meant to be free, including charges for vaccines, syringes and the child health card. A 30 year old mother of four emphasized:

*“For me what hurt me was when the (service providers) asked for money for the child health card. That money really hurt me.”* FGD women

Transport costs to immunisation facilities were incurred by 44% (95% CI 41–48) of the survey respondents. Respondents who utilised public facilities (72%, 95% CI 67–76) incurred transport costs more often than users of private facilities (28%, 95% CI 23–32) but this difference was not statistically significant (OR 1.32, 95%CI 0.83-2.08). A third (30%, 95% CI 26-33%) of the survey respondents reported that transport costs would deter them from seeking immunisation services. After multivariable analysis in this study, none of the barriers from the WHO building blocks were independent predictors of use of public or private facilities. Only the cost of immunisation had a borderline association with use of public or private immunisation facilities, Table 1 (Model *X*^2^ = 106.67; df = 9; p = 0.001).

## Discussion

Health system barriers identified in this urban setting were in service delivery, financing, human resources and vaccines and supplies. Some of the study participants lived far from immunisation facilities, which meant spending money on transport. Vaccinations are supposed to be free, but this study reports irregular costs that were not accounted for. Most public and almost all private facilities charged money for immunisation. The combined effect of distant facilities and few service providers resulted into long queues at the immunisation facilities and long waiting times before receipt of services at public facilities. Safety concerns for the child, rude service providers, and unqualified workers were major concerns for consumers. Service provision was further hindered by the lack of transport and vaccine shortages. These barriers led to cessation of or delays in childhood immunisations for some consumers.

Poor geographical access to services has been commonly reported in rural [31-33] but rarely in urban [18] settings. In Kampala, poor geographical access to services was previously addressed by engaging private facilities in immunisation service provision. However, we found that their involvement was still low, as demonstrated by the larger proportion of consumers utilizing public than private facilities for immunisation. Similar findings were reported from research on childhood diarrhoea and pneumonia [34], emphasizing the need for increased public-private partnerships for child health services. It has been demonstrated that public-private partnerships increase coverage of essential interventions for child survival in some places [35].

The low involvement of the private sector in immunisation service provision in Kampala could be explained by two main factors. First, the lack of financial support for immunisation activities at these facilities meant that the proprietors had to find additional resources to provide services. This might have led to the rampant informal charges for immunisations. Secondly, there was a reported lack of technical capacity for immunisation service provision, as evidenced by reports that unqualified health workers were engaged. This was also mentioned as underlying the consumers’ preference for public rather than private facilities, especially among participants in focus group discussions. Similar sentiments were expressed by consumers in a high resource urban setting, although the reason for their preference differed as they explained that vaccines were used more frequently in public clinics therefore the quality would be better [17]. The engagement of the private sector in service provision should therefore be a continuous activity and the dynamics need to be examined and redefined regularly.

The preference for immunisation from public facilities in Kampala was tempered by other factors that affected the quality of services such as long waiting times. As in other studies [36,37] long waiting times were reported to reduce service quality. Half of our participants were willing to wait for only up to an hour before receipt of services. There could be several reasons for the long waiting times, including having few facilities providing immunisation services, and having few health workers to provide services to the large number of consumers who turn up at the facility. On the other hand, the waiting times at some immunisation facilities in Kampala were long because health workers reported late at the work stations. The late reporting could be a reflection of low health worker motivation. Similar reports have been cited by other researchers in Uganda where health workers report late at the work stations and leave early because of low motivation [38]. The delayed allowances for immunisation activities aggravated the situation in Kampala since the delay has the potential to reduce health worker motivation [15] and could also lead to rampant informal charges for immunisation. Informal payments in our study were defined as payments to individuals or to institutions, in cash or in kind, that should have been covered by the health system [39]. Informal payments for health services were reported in another Ugandan district [38] and were cited as a barrier to immunisation especially for FGD participants in our study. Financial barriers could reduce vaccination rates by 10 to 15% [40]. In a Hong Kong study, the provision of free services was mentioned by many as a primary reason for high immunisation compliance [17].

Our study indicated lack of resources as a major hindrance to immunisation services. Shortages were cited in human resource, transport and supplies and vaccines. Some authors argue that the provision of resources does not necessarily translate into a positive implementation process [41]. We argue that resources are essential for improving immunisation programmes especially in this setting where they are lacking. This argument is supported by similar findings from a low income urban population in the USA, where having access to paediatric vaccination providers was associated with better immunisation status [18]. In addition, a study that explored the work environment of mid-level healthcare providers in Malawi revealed that inadequate resources in the work environment correlated with job dissatisfaction, dissatisfaction with the profession, and thinking about leaving one’s job [42]. The reported high attrition of health professionals especially in private facilities in Kampala could partly explain the shortage in immunisation service providers in Kampala.

Lack of transport and shortage of vaccine supplies were reported in both private and public facilities in Kampala. Lack of vaccines has been reported to affect service utilization negatively [43]. Similar to findings in our study, an assessment by GAVI revealed that vaccine shortages were due to irregular delivery and stocks tended to be below target amounts at national, district and primary health care levels [44].

Although there is ongoing research on the Ugandan health system [31], there has been little focus on immunisation services, a vital programme for child survival. Successful implementation of immunisation programmes requires adequate availability of all the items outlined in all the building blocks of the WHO health system framework. Deficiency in these could translate into poor health outcomes such as poor responsiveness, lack of social and financial risk protection, inefficient health systems and ultimately lack of improvement in population health [22]. With the 2015 deadline for the MDGs fast approaching, it is necessary for Uganda and other sub-Saharan African countries to assess their position critically and develop locally relevant strategies to overcome the barriers identified [7]. For example, waiting times at facilities could be improved through recruitment of additional health providers, training of more immunisation service providers to assist during immunisation days, and increasing the participation of private facilities in service provision. Greater involvement of the private sector could decongest the public facilities, which have long queues and therefore long waiting times. Available literature shows few successful interventions targeting immunisation services [12] emphasizing the need for further research to identify innovative and effective strategies for improving immunisation services.

### Strengths and limitations

One limitation of this study was that in the survey we interviewed only child caretakers with child health cards. Children without cards are at higher risk of not being vaccinated owing to health system barriers and this could have led to an underestimate of immunisation system barriers. However, the proportion of those without cards was relatively small (9%), and they shared demographic characteristics with the population analysed [25]. One of the strengths of this study was that FGDs and KIIs were held within the community, which included participants who were unable to overcome immunisation system barriers. Another study strength was that combining provider and consumer perspectives provided for building a more complete picture of the barriers to immunisation service delivery. Also, mixing quantitative and qualitative data in a concurrent design enabled us to use the strengths of both quantitative and qualitative analysis techniques to elucidate the barriers to immunisation services and to enhance the quality of data interpretation [40]. Our findings could be compared to urban and peri-urban settings in Uganda and similar settings in sub-Saharan Africa, but may not be comparable to rural communities where we estimate that barriers to immunisation services are more common.

## Conclusions

Much of the literature on immunisation from developing countries focuses on rural health systems where outreaches act as avenues for increasing access to immunisation through reducing distance to services for the caretakers. This study focused on urban immunisation contributing to the thinking that even in urban areas with great physical access, there are hard to reach categories of people. As the rate of urbanization increases in all continents especially in sub-Saharan Africa [45], this sounds a caution to governments to strengthen health systems to cater for the increasing urban populations. We have suggested resource-demanding efforts that could improve services, acknowledging the need for implementation research to fully capture how this would translate into improved vaccination coverage and ultimately improved immunity among children. In this paper we have also highlighted the importance of the public – private partnerships which are currently being promoted for other child health programmes [35]. For a long time, governments and other stakeholders have focused only on public health systems and the private system has been seen as disorganized and unregulated [46]. In this paper we explain how the private sector can actually be used to increase coverage for public goods like immunisation. We also highlighted the gaps that remain in the health system following inclusion of the private providers. Thus engagement with the private sector should be a continuous activity with regular evaluation of the process. Lastly, interventions that address the identified barriers to immunisation in Kampala could improve coverage by double digits; therefore these interventions should be a priority.

## Competing interests

The authors declare that they have no competing interests.

## Authors’ contributions

JNB, IMSE, and FN contributed to the conception and design of the study, data analysis, interpretation of data and drafting the paper. ER and JK contributed to data analysis, interpretation of data and drafting the paper. All authors approved the final manuscript.

## Pre-publication history

The pre-publication history for this paper can be accessed here:

http://www.biomedcentral.com/1472-6963/14/111/prepub
